# Robotics and Artificial Intelligence in Endovascular Neurosurgery

**DOI:** 10.7759/cureus.23662

**Published:** 2022-03-30

**Authors:** Javier Bravo, Arvin R Wali, Brian R Hirshman, Tilvawala Gopesh, Jeffrey A Steinberg, Bernard Yan, J. Scott Pannell, Alexander Norbash, James Friend, Alexander A Khalessi, David Santiago-Dieppa

**Affiliations:** 1 Neurological Surgery, University of California San Diego, San Diego, USA; 2 Engineering and Healthcare Technology, University of California San Diego Jacobs School of Engineering, San Diego, USA; 3 Department of Neurology, Royal Melbourne Hospital, Melbourne, AUS

**Keywords:** ai and machine learning, computer-assisted diagnosis, neurosurgery, endovascular, robotics, artificial intelligence

## Abstract

The use of artificial intelligence (AI) and robotics in endovascular neurosurgery promises to transform neurovascular care. We present a review of the recently published neurosurgical literature on artificial intelligence and robotics in endovascular neurosurgery to provide insights into the current advances and applications of this technology.

The PubMed database was searched for "neurosurgery" OR "endovascular" OR "interventional" AND "robotics" OR "artificial intelligence" between January 2016 and August 2021. A total of 1296 articles were identified, and after applying the inclusion and exclusion criteria, 38 manuscripts were selected for review and analysis. These manuscripts were divided into four categories: 1) robotics and AI for the diagnosis of cerebrovascular pathology, 2) robotics and AI for the treatment of cerebrovascular pathology, 3) robotics and AI for training in neuroendovascular procedures, and 4) robotics and AI for clinical outcome optimization.

The 38 articles presented include 23 articles on AI-based diagnosis of cerebrovascular disease, 10 articles on AI-based treatment of cerebrovascular disease, two articles on AI-based training techniques for neuroendovascular procedures, and three articles reporting AI prediction models of clinical outcomes in vascular disorders of the brain. Innovation with robotics and AI focus on diagnostic efficiency, optimizing treatment and interventional procedures, improving physician procedural performance, and predicting clinical outcomes with the use of artificial intelligence and robotics. Experimental studies with robotic systems have demonstrated safety and efficacy in treating cerebrovascular disorders, and novel microcatheterization techniques may permit access to deeper brain regions. Other studies show that pre-procedural simulations increase overall physician performance. Artificial intelligence also shows superiority over existing statistical tools in predicting clinical outcomes.

The recent advances and current usage of robotics and AI in the endovascular neurosurgery field suggest that the collaboration between physicians and machines has a bright future for the improvement of patient care. The aim of this work is to equip the medical readership, in particular the neurosurgical specialty, with tools to better understand and apply findings from research on artificial intelligence and robotics in endovascular neurosurgery.

## Introduction and background

Artificial intelligence (AI) is generally defined as the ability of a machine to analyze data and use it to learn and model human behavior. The general subtypes of AI technology that are currently being used and studied in healthcare are machine learning (ML), which identifies and analyzes patterns to detect associations in a dataset; deep learning (DL), which allows machines to make decisions through the use of neural network (NN) models; natural language processing, which allows machines to analyze human language; computer vision, through which computers can learn by analyzing images and videos; and physical robotics used for surgical procedures [1,2]. In the last 10 years, there has been an exponential increase in the study and use of AI in the medical field due in part to the ever-growing datasets that can be used to train and test emerging NNs and algorithms [3]. AI has demonstrated the potential to improve diagnostic accuracy, predict clinical outcomes, increase precision in surgical procedures, accelerate decision-making, and aid physicians in selecting ideal treatment protocols.

By comparison, robotic systems in surgery are generally divided into three categories: 1) active systems, which work autonomously; 2) semi-active systems, in which the surgeon complements the preprogrammed component of the robot; and 3) surgeon-driven systems [4]. The current robotic systems utilized in endovascular surgery fall into the surgeon-driven mechanism. They consist of a mechanical robot on the patient side and an operator control station that is radiation-shielded and allows the operator to control catheters and guidewires via sensors and joysticks [5]. Advantages to robotic systems in endovascular procedures include improved stability of the catheter tip, which in turn decreases the number of movements needed, enhanced performance navigating through tortuous vascular anatomy, and decreased exposure to radiation [6,7].

Understanding the impact and advances that robotics and AI have in the overall management of cerebrovascular disease will fundamentally and permanently enhance endovascular neurosurgery. Artificial intelligence has shown promise in both diagnostic and treatment settings. Deep learning algorithms have been effective in image recognition and may be instrumental in the improvement of decision-making in the clinical setting [8,9]. For example, Titano et al. demonstrated that a DL algorithm was capable of interpreting and triaging urgent neurological findings on head computed tomographies (CTs) 150 times faster than humans in the setting of intracranial hemorrhage (ICH), stroke, and hydrocephalus [10], and similar studies have shown encouraging results in stroke imaging and stroke care [11,12]. Physical robotic systems, such as the da Vinci robotic system (Intuitive Surgical Inc., Sunnyvale, CA, USA), have also become broadly accepted and proven to boost surgical precision, especially in abdominal surgery and prostatectomies.

While the use of robotics in neurovascular intervention is in its infancy, promising steps have been made to incorporate this technology into clinical practice. Interventionalists have employed AI and robotics to enhance medical treatment, yet most advances have been implemented in interventional cardiology; for instance, the CorPath GRX robotic system (Corindus, Waltham, MA, USA) has been utilized for coronary interventions since 2012 and was approved for peripheral vascular procedures in 2018 [13]. The CorPath GRX is also widely used in endovascular neurosurgery for procedures such as endovascular coiling and has strong potential for optimizing stroke thrombectomy. The FDA approved the Magellan Robotic System (Hansen Medical, Auris Health, Redwood, CA, USA) for clinical use in 2012 [5], and this system has also shown promise in neurovascular procedures, especially in the treatment of carotid artery stenosis (CAS) [14,15]. These robotic systems help control the catheter and augment support during interventional procedures [13]. Experiments have also been conducted on robotic endovascular devices and catheters that may allow for easier endovascular navigation and access to deeper regions in the brain [16-18]. A number of DL and ML algorithms have also been studied in the context of cerebrovascular disease. For example, the Viz large vessel occlusion (LVO) deep learning neural network (DLNN) has shown promising results in stroke care to reduce diagnosis and treatment times [19]. Similar studies have evaluated the use of other ML and DL frameworks for the optimization of the diagnosis of stroke and large vessel occlusion (LVO), intracranial (IC) aneurysms, intracranial hemorrhage (ICH), and other pathologies.

Within this work, we review the recent literature on the role of AI and robotics in endovascular neurosurgery to summarize significant findings from a series of reports and experimental studies in order to inform neurosurgeons and physicians in any medical specialty who wish to better understand emerging technology and its influence on clinical practice. Specifically, we survey the literature from January 2016 to August 2021 to understand how AI and robotics impact the diagnosis of cerebrovascular disorders, the treatment of cerebrovascular disorders, the training of endovascular neurosurgeons, and the outcome optimization in the management of neurovascular disease.

## Review

Methods

In order to perform an analysis of the existing literature on robotics and artificial intelligence in endovascular neurosurgery, the PubMed database was searched for articles between January 2016 and August 20, 2021. We selected this time window to focus on the examination of the most recent literature. The search terms utilized were "neurosurgery" OR "endovascular" OR "interventional" AND "robotics" OR "artificial intelligence." Initially, 1296 articles were identified.

Articles were included in this study if they presented primary data related to AI or robotics in the context of endovascular neurosurgery (i.e., treatment and diagnosis of intracranial aneurysms, arteriovenous malformations (AVM), arteriovenous fistulas, intracranial hemorrhage, and strokes), as well as optimization of catheterization procedures. To focus on novel primary data, literature reviews were excluded. To focus further on endovascular neurosurgery, articles were excluded if their focus was on non-neurovascular pathology (e.g., brain tumors and hydrocephalus).

A careful reading of the above articles revealed four general classes of AI and robotics research: 1) robotics and AI for diagnosis in endovascular pathology, 2) robotics and AI for the treatment of endovascular pathology, 3) robotics and AI for training in neuroendovascular procedures, and 4) robotics and AI for clinical outcome optimization. Articles under the diagnosis category were further classified into intracerebral hemorrhage (ICH) and Moyamoya disease detection, vascular visualization and diagnosis of vascular pathology (i.e., angiography, AVMs, aneurysms, and AV fistulas), and diagnosis of large vessel occlusion (LVO) and stroke. This subclassification is intended to help the reader map the many uses of AI as they pertain to different cerebrovascular pathologies.

Results

**Figure 1:**
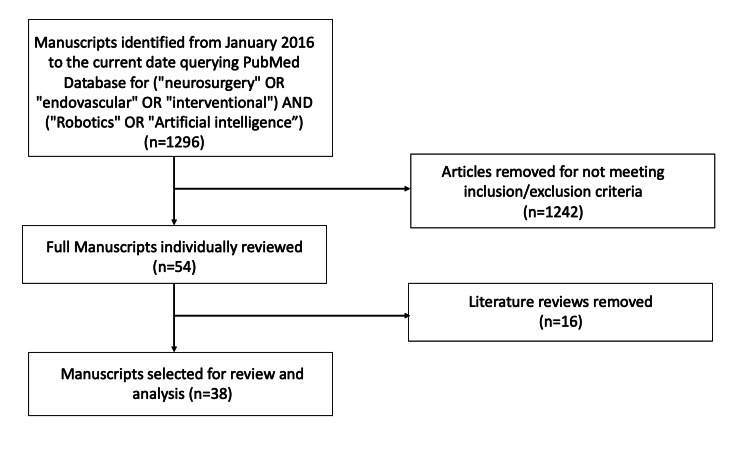
Article selection flowchart.

A total of 1296 articles were initially identified from the PubMed database. After applying the inclusion and exclusion criteria, 38 papers were selected for review and analysis: 23 articles on AI-based diagnosis of cerebrovascular disease, 10 articles on AI-based treatment of cerebrovascular disease, two articles on AI-based training techniques for neuroendovascular procedures, and three articles reporting AI prediction models of clinical outcomes in vascular disorders of the brain. Figure 1 shows a flowchart representing the article selection process.

AI-Based Diagnosis of Cerebrovascular Disorders

Twenty-three articles describing AI-based diagnostics in endovascular neurosurgery were included. A complete description of the results from these studies can be found in Table 1. Within the 23 articles, we identified 12 retrospective reviews, five retrospective cohort studies, one prospective cohort study, one prospective observational study, one cross-sectional retrospective review, two experimental studies, and one case series study.

We identified seven articles that demonstrated the potential clinical utility and time-effectiveness that DL algorithms offer in the setting of stroke and LVO [19-25]. Morey et al. demonstrated that the Viz LVO DLNN reduces the door-to-neuroendovascular team notification time in patients with acute ischemic stroke [19]. Yahav-Dovrat et al. also utilized the Viz LVO DLNN as a stroke diagnostic tool but reported a high false-positive rate (66%) [20]. Block et al. demonstrated a more time-effective identification of cerebral ischemia signs through neural network-processed physiological and clinical data [21]. An ML algorithm was also capable of distinguishing patients with basal ganglia stroke onset of 4.5 hours or more by CT scan analysis in a retrospective review by Yao et al. [22]. Rava et al. and Bernard et al. both established that DL CT image processing is accurate in stroke diagnosis [23,24]. Kasasbeh et al. also tested an artificial NN that used computed tomography perfusion images and baseline clinical data to successfully predict the ischemic core in stroke patients [25].

Seven of the identified studies focused on intracranial aneurysm detection and surveillance and reported successful results using AI-assisted diagnosis [26-32]. Silva et al. found that an ML algorithm can classify aneurysm rupture status based on previously established predictors [26]. Zhu et al. evaluated an ML model that exhibited superior performance in assessing intracranial aneurysm stability than an existing statistical logistical regression model and the population, hypertension, age, size of aneurysm, earlier subarachnoid hemorrhage (SAH) from another aneurysm, and site of aneurysm (PHASES) score that predicts an absolute five-year risk of intracranial aneurysm rupture [28]. Park et al. evaluated the HeadXNet DLNN and found that physicians who utilized this AI tool had an improved sensitivity, accuracy, and interrater reliability in the diagnosis of intracranial aneurysms [30]. Shimada et al. demonstrated that a NN model was capable of detecting unruptured intracranial aneurysms measuring <2 mm in diameter that were previously missed by two radiologists [29]. Kim et al. described an AI prediction model that found that aneurysm size has the most significant influence on the risk of vasospasm in the setting of aneurysmal subarachnoid hemorrhage (SAH) [32]. A prospective cohort study conducted by Kordzadeh et al. determined that a DLNN model could predict the functional maturation of AV fistula with >80% accuracy [33]. Two articles evaluated AI in imaging processing [34,35]. Fu et al. assessed a DLNN (CerebralDoc) for computed tomography angiography reconstruction, reporting high image quality and significantly reduced postprocessing times [34]. Lang et al. proved that AI-based 3D angiography is a reliable method for visualization of cerebral vasculature that additionally might reduce radiation exposure to the patient [35]. Heunis et al. explored a robotic ultrasound system and revealed a conceivable radiation-free alternative to catheterization procedures in an animal model [36].

Four studies were related to AI-based ICH diagnosis or classification [37-40], and one study evaluated the diagnosis of Moyamoya disease [41]. Teng et al. reported that a DL model, BioMind (BioMind Technologies, Beijing, China), has high sensitivity and specificity for the early prediction of ICH expansion [38]. Rava et al. found that the DL model AUTOStroke Solution Program (Canon Medical Systems, Otawara, Japan) demonstrated effectiveness in detecting ICH with volumes > 3 mL [37]. Nawabi et al. found that an ML algorithm was capable of accurately distinguishing between neoplastic and non-neoplastic ICH [39]; similar findings have been reported by Yeo et al. [42]. Another DL algorithm explored by Voter et al. interestingly demonstrated decreased diagnostic accuracy in the setting of ICH [40]. Akiyama et al. tested a DL algorithm to differentiate between Moyamoya disease and atherosclerosis by analyzing T2-weighted images, reporting high accuracies to do so in the basal cistern, basal ganglia, and centrum semiovale levels [41].

**Table 1:** Twenty-three studies on AI/robotics for the diagnosis of cerebrovascular disorders.

Author	Year	Type of study	Title	Time	Sample size	AI/robotics subtype	Key objective	Key findings
Akiyama et al. [41]	2020 (September)	Retrospective review	Deep Learning-Based Approach for the Diagnosis of Moyamoya Disease	2009 to 2016	84	Deep learning algorithm	Moyamoya disease diagnosis	AI analyzing T2-weighted images showed high-accuracy results in distinguishing between atherosclerotic disease and Moyamoya disease at the level of the basal cistern, basal ganglia, and centrum semiovale.
Kordzadeh et al. [33]	2019 (March)	Prospective cohort study	The Role of Artificial Intelligence in the Prediction of Functional Maturation of Arteriovenous Fistula	2012 to 2016	266	Deep learning neural network model	AV fistula maturation prediction	With 10 given patient attributes, AI could predict functional maturation of AV fistula with >80% accuracy (p < 0.01).
Lang et al. [35]	2020 (October)	Retrospective review	Evaluation of an Artificial Intelligence-Based 3D-Angiography for Visualization of Cerebral Vasculature	2019	15	Deep learning neural network model	Cerebral angiography optimization	An AI-based 3DA technique based only on a single contrast-enhanced run that functions with approximately half of the radiation required for the conventional subtraction technique shows comparable results to standard 3D DSA with a significant reduction in patient radiation dose.
Silva et al. [26]	2019 (November)	Retrospective cohort study	Machine Learning Models can Detect Aneurysm Rupture and Identify Clinical Features Associated with Rupture	2002 to 2018	615	Machine learning algorithm	Aneurysm rupture detection	The model can accurately classify aneurysm rupture status based on previously established predictors. The model suggests that location is significantly more important than size when estimating rupture risk. The ML techniques show promise in clinical neurosurgical applications.
Faron et al. [27]	2019 (June)	Retrospective review	Performance of a Deep-Learning Neural Network to Detect Intracranial Aneurysms from 3D TOF-MRA Compared to Human Readers	2015 to 2017	85	Deep learning neural network model	IC aneurysm diagnosis	Statistical analysis revealed no significant differences in overall sensitivity between the neural network, reader 1, and reader 2. Human readers detected a significantly higher portion of aneurysms (<3 mm) compared to the neural network in this study. In a clinical setting, neural network algorithms may potentially increase detection rates of cerebral aneurysms.
Zhu et al. [28]	2020 (May)	Retrospective review	Stability Assessment of Intracranial Aneurysms Using Machine Learning Based on Clinical and Morphological Features	2014 to 2018	1897	Machine learning random forests (RF) and support vector machine (SVM) and automated neural network	IC aneurysm diagnosis	ML models displayed better performance than the statistical LR model and PHASES score in intracranial aneurysm stability assessment.
Shimada et al. [29]	2020 (October)	Case series	Incidental cerebral aneurysms detected by a computer-assisted detection system based on artificial intelligence	2017 to 2018	1623	Convolutional neural network	IC aneurysm diagnosis	A neural network model and computer-assisted diagnosis detected five unruptured intracranial aneurysms measuring <2 mm in diameter previously missed by two radiologists.
Park et al. [30]	2019 (June)	Retrospective review	Deep Learning-Assisted Diagnosis of Cerebral Aneurysms Using the HeadXNet Model	2003 to 2017	9455	Deep learning neural network model (HeadXNet)	IC aneurysm diagnosis	The DL model was successful in detecting intracranial aneurysms on CTA, and physicians using the model as aid had an improved sensitivity, accuracy, and interrater reliability in the diagnosis of intracranial aneurysms.
Liu et al. [31]	2021 (March)	Cross-sectional retrospective review	Deep neural network-based detection and segmentation of intracranial aneurysms on 3D rotational DSA	2014 to 2018	451	Deep learning neural network (3D-Dense-UNet Model)	IC aneurysm diagnosis	The combination of the 3D-Dense-UNet model and 3D RA images may have a high sensitivity in the detection of intracranial aneurysms with a low false-positive rate.
Fu et al. [34]	2020 (September)	Retrospective review	Rapid vessel segmentation and reconstruction of head and neck angiograms using 3D convolutional neural network	2017 to 2018	18766	Deep learning neural network (CerebralDoc)	Cerebral angiography optimization	DL (CerebralDoc) offers an efficient and fast method to reconstruct head and neck CTAs compared to currently utilized techniques. It may save costs and increase efficiency in radiology daily clinical workflow.
Kim et al. [32]	2021 (September)	Retrospective review	Analysis of risk factors correlated with angiographic vasospasm in patients with aneurysmal subarachnoid hemorrhage using explainable predictive modeling	2011 to 2019	343	Machine learning	SAH vasospasm risk analysis	According to the AI prediction model, aneurysm size has the most significant influence on the risk of vasospasm in the setting of aneurysmal SAH.
Teng et al. [38]	2021 (May)	Retrospective cohort study	Artificial Intelligence Can Effectively Predict Early Hematoma Expansion of Intracerebral Hemorrhage Analyzing Noncontrast Computed Tomography Image	2011 to 2018	118	Deep learning neural network (BioMind)	ICH sizing	The sensitivity of intracerebral hemorrhage hematoma expansion predicted by the artificial intelligence imaging system was found to be 89.3%, with a specificity of 77.8%, a positive predictive value of 55.6%, a negative predictive value of 95.9%, and a Yoden index of 0.671.
Rava et al. [37]	2021 (June)	Retrospective cohort study	Assessment of an Artificial Intelligence Algorithm for Detection of Intracranial Hemorrhage	2016 to 2019	302	Deep learning algorithm (AUTOStroke Solution)	ICH diagnosis	The ICH detection algorithm was capable of detecting IPHs, IVHs, SDHs, and SAHs accurately, as well as determining the absence of ICH.
Nawabi et al. [39]	2020 (May)	Retrospective review	Neoplastic and Non-neoplastic Acute Intracerebral Hemorrhage in CT Brain Scans: Machine Learning-Based Prediction Using Radiomic Image Features	2010 to 2017	77	Machine learning	ICH classification	The ML approach employing quantitative image features derived from non-contrast-enhanced CT scans provides high discriminatory accuracy in predicting neoplastic ICHs.
Voter et al. [40]	2021 (April)	Retrospective review	Diagnostic Accuracy and Failure Mode Analysis of a Deep Learning Algorithm for the Detection of Intracranial Hemorrhage	2019	3605	Deep learning algorithm (Aidoc, Aidoc Medical, Tel Aviv, Israel)	ICH diagnosis	The use of AI diagnostic tool demonstrated decreased diagnostic accuracy compared to current methods, emphasizing the need for standardized study designs.
Morey et al. [19]	2021 (April)	Retrospective review	Real-World Experience with Artificial Intelligence-Based Triage in Transferred Large Vessel Occlusion Stroke Patients	2018 to 2020	55	Deep learning neural network (Viz LVO)	Stroke diagnosis optimization	Implementation of the Viz LVO model in the management of large vessel occlusion acute ischemic stroke patients transferred for endovascular therapy is associated with decreased door-to-neuroendovascular team notification time intervals.
Bernard et al. [24]	2021 (January)	Retrospective cohort study	Deep learning reconstruction versus iterative reconstruction for cardiac CT angiography in a stroke imaging protocol: reduced radiation dose and improved image quality	2018 to 2019	296	Deep learning neural network	Stroke diagnosis optimization	DLR for cardiac CT angiography in an acute stroke imaging protocol improved the image quality and reduced the radiation dose compared to the use of iterative reconstruction.
Rava et al. [23]	2021 (March)	Retrospective cohort study	Validation of an artificial intelligence-driven large vessel occlusion detection algorithm for acute ischemic stroke patients	2019 to 2020	303	Deep learning algorithm (AUTOStroke Solution)	Stroke diagnosis optimization	The DL algorithm was capable of recognizing ICA and M1 MCA occlusions with precision. It also was highly accurate in ruling out large vessel occlusion but had a lower sensitivity for detecting M2 and MCA occlusions.
Block et al. [21]	2020 (June)	Prospective observational study	Cerebral ischemia detection using artificial intelligence (CIDAI)—A study protocol	2020	20 (ongoing)	Convolutional neural network	Stroke diagnosis optimization	Physiological and clinical data processed by AI could be used to more rapidly identify early signs of cerebral ischemia.
Yao et al. [22]	2020 (May)	Retrospective review	CT radiomics features as a diagnostic tool for classifying basal ganglia infarction onset time	2016 to 2019	316	Machine learning algorithm	Stroke diagnosis optimization	Patients with stroke onset within 4.5 hours or more could be distinguished by image analysis based on CT scans.
Yahav- Dovrat et al. [20]	2021 (February)	Retrospective review	Evaluation of Artificial Intelligence-Powered Identification of Large-Vessel Occlusions in a Comprehensive Stroke Center	2018 to 2019	1167	Deep learning neural network (Viz LVO)	Stroke diagnosis optimization	The Viz LVO algorithm demonstrated high accuracy but had a false-positive rate of 66%. The system has potential for the early detection of patients with stroke but requires improvements to establish a higher accuracy.
Kasasbeh et al. [25]	2019 (May)	Experimental study	Artificial Neural Network Computer Tomography Perfusion Prediction of Ischemic Core	2019	128	Artificial neural network	Stroke diagnosis optimization	The artificial neural network incorporated with computed tomography perfusion and clinical data was able to accurately predict ischemic core in stroke patients.
Heunis et al. [36]	2021 (November)	Experimental study	Real-Time Multi-Modal Sensing and Feedback for Catheterization in Porcine Tissue	2020	N/A	Robotic system	Catheterization optimization	An autonomous ultrasound robotic system equipped with a multi-modal sensing and feedback framework enables radiation-free and accurate reconstruction of significant tissues and instruments in catheterization procedures.

AI-Based Treatment of Cerebrovascular Disorders

Ten articles describe the role of AI/robotics-based treatment of neurovascular disorders [14-18,43-47]. Table 2 shows a description of the results of these studies. We included six experimental studies, one retrospective evaluation, one retrospective cohort study, one case report, and one technical report. Endovascular procedure optimization was the key objective in all of these studies. Both physical robotics and advancements in catheterization were explored. Pancaldi et al. introduced a flow-driven endovascular navigation device that may technically permit access to deeper brain regions through the use of interventional robotics [16]. Similarly, Gopesh et al. tested and explored a steerable endovascular microcatheter that could facilitate access and treatment of deep intracranial aneurysms [17]. Bao et al. explored a novel remote-controlled vascular interventional robot (RVIRC), which demonstrated the accurate operation of a catheter and guidewire, findings that suggest its usefulness in catheterization procedures [18]. Britz et al. explored modifications to the CorPath GRX system that could further improve its performance and effectiveness in the catheterization of neurovascular anatomy [43], while George et al. and Nogueira et al. demonstrated that the CorPath GRX Robotic System is a feasible, safe, and effective alternative in the treatment of carotid artery stenosis (CAS) [15,47]. Jones et al. had a similar goal and explored a different vascular interventional system, the Magellan Robotic System, in carotid artery stenting, demonstrating its safety and effectiveness even in the setting of complex vascular anatomy [14]. Several papers addressed one of the common criticisms of robotic catheterization: the lack of sensation and haptic feedback, which is crucial to the interventionalist during catheterization procedures. Miyachi et al. tested a force-sensing feedback framework that alerts the operator via an audible scale to try to address this problem; the results were promising for future adaptations of this technology [46]. Another modification to endovascular robotic systems was evaluated by Chi et al. to try to incorporate three-dimensional preoperative imaging into the robotic platform. This resulted in a 33.3% reduction in mean contact forces with smoother catheter paths [45].

**Table 2:** Ten studies on AI/robotics for the treatment of cerebrovascular disorders.

Author	Year	Type of study	Title	AI/robotics subtype	Key objective	Key findings
Pancaldi et al. [16]	2020 (December)	Experimental study	Flow driven robotic navigation of microengineered endovascular probes	Robotic endovascular navigation device	Endovascular procedure optimization	Using this technology, endovascular access to deep brain regions is technically feasible.
Britz et al. [43]	2019 (November)	Experimental study	Neuroendovascular-specific engineering modifications to the CorPath GRX Robotic System	Vascular interventional robot (CorPath GRX Robotic System)	Endovascular procedure optimization	Modifications to the CorPath GRX Robotic System previously used for cardiac and peripheral vascular interventions allow improved effectiveness in neurovascular anatomy.
Jones et al. [14]	2021 (January)	Prospective evaluation	Robot-Assisted Carotid Artery Stenting: A Safety and Feasibility Study	Vascular interventional robot (Magellan Robotic System)	Endovascular procedure optimization	Endovascular robotic carotid artery stenting is safe and effective, demonstrating success even in the setting of challenging anatomy.
Bao et al. [18]	2018 (February)	Experimental study	A cooperation of catheters and guidewires-based novel remote-controlled vascular interventional robot	Vascular interventional robot (RVIR-CI)	Endovascular procedure optimization	The RVIR-CI was demonstrated to accurately operate a catheter and guidewire, detect resistance forces, and complete complex surgical procedures by cooperation between catheters and guidewires.
Cheung et al. [44]	2020 (October)	Retrospective cohort study	Comparison of manual versus robot-assisted contralateral gate cannulation in patients undergoing endovascular aneurysm repair	Vascular interventional robot (Magellan Robotic System)	Endovascular procedure optimization	Utilizing a vascular interventional robot for contralateral gate cannulation in endovascular aneurysm repair resulted in decreased navigation path lengths and increased economy of movement compared to manual techniques. Robotic catheterization also showed increased cannulation times.
Chi et al. [45]	2018 (April)	Experimental study	Learning-based endovascular navigation through the use of non-rigid registration for collaborative robotic catheterization	Learning from demonstration (LfD)-equipped vascular interventional robot	Endovascular procedure optimization	Incorporating three-dimensional preoperative imaging into a semiautonomous robotic catheterization platform was associated with smoother and shorter path lengths, as well as less mean and maximum contact forces than a manual approach.
Gopesh et al. [17]	2021 (August)	Experimental study	Soft robotic steerable microcatheter for the endovascular treatment of cerebral disorders	Hydraulically actuated soft robotic steerable tip at dimensions compatible with cerebral arteries	Endovascular procedure optimization	The microcatheter was successfully steered in a pig model, and the deployment of coils in complex vascular anatomy was successful.
George et al. [15]	2020 (May)	Case report	Robotic-assisted balloon angioplasty and stent placement with distal embolic protection device for severe carotid artery stenosis in a high-risk surgical patient	Vascular interventional robot (CorPath GRX Robotic System)	Endovascular procedure optimization	The CorPath GRX endovascular robotic system was successfully used in the placing of balloons and stents for the treatment of severe carotid artery stenosis.
Miyachi et al. [46]	2021 (May)	Experimental study	Remote Surgery Using a Neuroendovascular Intervention Support Robot Equipped with a Sensing Function: Experimental Verification	Vascular interventional robot	Endovascular procedure optimization	A remote endovascular robotic system was tested using a force-measuring device for sensing feedback, yielding promising results for its use in neurovascular treatment and procedures.
Nogueira et al. [47]	2020 (March)	Technical report	Robotic assisted carotid artery stenting for the treatment of symptomatic carotid disease: technical feasibility and preliminary results	Vascular interventional robot (CorPath GRX Robotic System)	Endovascular procedure optimization	Robotic-assisted carotid artery stenting is feasible and safe. All steps of the procedure were performed with success, except for stent navigation and deployment.

AI/Robotics in Neuroendovascular Training

Two studies looking at AI/robotics-based training and simulation of neurovascular procedures were included [48,49]. A full description of the results can be found in Table 3. Yamaki et al. evaluated a simulation technique involving endovascular robotics and a flow-driven robotic stent [48]. An experimental study by Pannell et al. tested the ANGIO Mentor Simulator (Simbionix, Cleveland, OH, USA) and found that simulated procedures produced a significant performance improvement in angiograms, embolectomies, and aneurysm coil embolizations by neurosurgical residents and neuroradiology fellows [49]. Both studies illustrate the utility of pre-procedural rehearsal in endovascular neurosurgery and robot-assisted interventions and also demonstrate the need for further studies of this nature.

**Table 3:** Two studies on AI/robotics for neuroendovascular training.

Author	Year	Type of study	Title	AI	Key objective	Key findings
Yamaki et al. [48]	2021 (May)	Experimental study	Biomodex patient-specific brain aneurysm models: the value of simulation for first in-human experiences using new devices and robotics	Vascular interventional robot and flow-diverted stent	Assess the reliability of an experimental treatment rehearsal model	Pre-procedural rehearsal using patient-specific 3D models provides precise procedure planning, which can potentially lead to greater operator confidence, decreased radiation dose, and improvements in patient safety, particularly in first in-human experiences.
Pannell et al. [49]	2016 (August)	Experimental study	Simulator-Based Angiography and Endovascular Neurosurgery Curriculum: A Longitudinal Evaluation of Performance Following Simulator-Based Angiography Training	ANGIO Mentor Simulator	Establish performance metrics for angiography and neuroendovascular surgery procedures based on longitudinal improvement in individual trainees with differing levels of training and experience	Neurosurgical residents and neuroradiology fellows should perform a minimum of five simulated angiograms, five simulated embolectomies, and 10 simulated aneurysm permanent coil embolizations prior to scrubbing for endovascular neurosurgery cases. Participants demonstrated statistically significant performance improvements after performing simulations.

AI/Robotics in Clinical Outcome Optimization for Neurovascular Disease

Three articles described the use of AI in outcome prediction and optimization in the setting of neurovascular disease and treatment. Two retrospective studies and one prospective study were included [50-52]. Table 4 shows a full description of the results. Asadi et al. evaluated the effectiveness of an ML algorithm in predicting complications after endovascular treatment of brain arteriovenous malformations (BAVMs) and found it to have an accuracy of 43% in predicting mortality; it also showed a 97.5% accuracy in predicting outcomes and identified the presence or absence of nidal fistulae as the most important factor [50]. A similar study has also shown promise in ML for clinical outcome prediction in the setting of stroke [53]. Katsuki et al. demonstrated that AI prediction frameworks may be constructed with relative ease and have superior performance to existing statistical prediction models (SAFIRE score and Fisher CT scale) [51]. Finally, a neural network was evaluated by De Jong et al. for outcome prediction in the setting of aneurysmal SAH and reported sensitivity rates of 82% for mortality, 94% for unfavorable modified Rankin scales, and 74% for delayed cerebral ischemia (DCI) and specificity rates of 80%, 80%, and 68% for these same three outcomes, respectively [52].

**Table 4:** Three studies on AI and clinical outcome optimization.

Author	Year	Type of study	Title	Sample size	AI	Key objective	Key findings
Asadi et al. [50]	2016 (December)	Retrospective study	Outcomes and Complications After Endovascular Treatment of Brain Arteriovenous Malformations: A Prognostication Attempt Using Artificial Intelligence	199	Machine learning	Intracranial hemorrhage was the most common clinical presentation (56%); all spontaneous events occurred in previously embolized BAVMs remote from the procedure; the standard regression analysis model had an accuracy of 43% in predicting final outcome (mortality), with the type of treatment complication identified as the most important predictor	Machine learning techniques can predict final outcomes with greater accuracy and may help individualize treatment based on key predicting factors.
Katsuki et al. [51]	2021 (June)	Retrospective study	Easily Created Prediction Model Using Automated Artificial Intelligence Framework (Prediction One, Sony Network Communications Inc., Tokyo, Japan) for Subarachnoid Hemorrhage Outcomes Treated by Coiling and Delayed Cerebral Ischemia	298	Machine learning	Comparison of an AutoAI framework (Prediction One) and existing statistical prediction models (SAFIRE score and Fisher CT scale) for SAH outcomes	The AUCs of the AutoAI-based models for functional outcome in the training and test dataset were 0.994 and 0.801, respectively, and those for the DCI occurrence were 0.969 and 0.650, respectively. The AUCs for functional outcomes calculated using the modified SAFIRE score were 0.844 and 0.892. Those for the DCI occurrence calculated using the Fisher CT scale were 0.577 and 0.544. AutoAI could easily and quickly produce prediction models in less than two minutes as long as we provide the dataset.
De Jong et al. [52]	2021 (May)	Prospective study	Prediction Models in Aneurysmal Subarachnoid Hemorrhage: Forecasting Clinical Outcome With Artificial Intelligence	585	Machine learning	To investigate the prediction capacity of feedforward artificial neural networks (ffANNs) for the patient-specific clinical outcome and the occurrence of delayed cerebral ischemia (DCI) and compare those results with the predictions of two internationally used scoring systems	The presented ffANN showed equal performance when compared with the VASOGRADE and SAHIT scoring systems while using fewer individual cases.

Discussion

Artificial Intelligence and robotic systems have transformed the practice of medicine, allowing for time-effective diagnosis, efficient patient categorization and treatment, improved diagnostic accuracy, and precise and safe surgical interventions. We describe the spectrum of various AI algorithms and robotic systems in endovascular neurosurgery and give readers an opportunity to understand the current impact that this technology may have on neurovascular practice and patient care. In particular, ML and DL algorithms may facilitate the detection of life-threatening conditions such as stroke and LVO, ICH, and ruptured aneurysm, while other algorithms can assist with surveillance such as ensuring that unruptured aneurysms have not grown or changed in morphology. For ischemic stroke, Morey et al. used the Viz LVO algorithm in a real-world experiment, and it resulted in decreased door-to-neurovascular team notification times, which in turn resulted in faster reperfusion and augmented clinical outcomes [19]. Evolving algorithms also show promise in intracranial aneurysm and ICH detection and classification. These changes have exceeded human radiologist capability as radiologist augmentation with a DL algorithm resulted in the detection of a significantly higher proportion of intracranial aneurysms than by physicians alone [27]. This technology has the power and potential to improve with time.

Robotic systems in interventional procedures also have the potential for more practical interventions and improved outcomes. Although robotic systems are generally thought to decrease procedural times, Cheung et al. and Weinberg et al. have demonstrated that this is not always true, primarily owing to increased robotic cannulation times and a lack of operator familiarity with the robotic system [44,54]. However, learning curves are expected in every field, and as providers become more experienced with robotics, this trend may improve as has been shown in other domains of surgical medicine [55].

Robotics naturally may make surgery safer for providers as well. The Percutaneous Robotically Enhanced Coronary Intervention (PRECISE) study found that by using robotic aids, the median radiation exposure to operators was reduced by 95.2% (0.98 versus 20.6 mGy, p=0.001) [56]. The use of robotic systems may also diminish the orthopedic burden on operators since this would eliminate the need for heavy lead gowns during procedures as the operator would be comfortably sitting behind a radiation-protected cockpit. Novel enhancements to endovascular catheters and microcatheters may facilitate safe navigation through tortuous anatomy and allow for the treatment of aneurysms and other vascular pathology in deeper cerebral regions while exposing the surgeon to less radiation [16,17]. The enhanced catheter tip stability and smoother catheter and guidewire navigation that robotic systems offer could prompt an even steeper rise in the evaluation and adoption of this technology in neurovascular practice.

Although robotic systems in endovascular procedures have limitations such as a lack of haptic feedback, the literature in this review suggests that the incorporation of this technology is forthcoming and will continue to improve. In this landscape of rapid change and innovation, limitations to AI and robotics must be further explored and studied through rigorous testing. Although this poses a challenge for the development of a larger number of clinically safe algorithms, AI technology may pave the way for the future in clinical practice and augment practicing physicians’ capabilities to efficiently recognize life-threatening conditions.

As the incorporation of AI and robotics continue, this new technology will fundamentally have an impact on healthcare costs to the individual and society. Approximately $3.3 trillion is spent by the United States on healthcare [57,58], and the addition of robotic systems may increase the economic burden initially. Nevertheless, the improved clinical outcomes associated with robotics may lead to an increase in overall health and quality of life, as well as a reduction in long-term costs. The long-term impact of investing in this technology and its impact on healthcare costs must be further studied. For example, the CorPath GRX System developed by Corindus has an initial capital cost of approximately $500K with an additional $400-$750 cost per procedure, and the Magellan Robotic System is estimated at around $1 million. However, if these modalities improve outcomes, the costs of innovation are justified [13]. One study estimated that the instruments and accessories used in robotic surgery cost an average of $1866 per procedure [59]. Despite the ongoing study and approval of AI and robotics in clinical medicine, few analyses detailing their cost-effectiveness have been made thus far in the field of endovascular neurosurgery [60]. Additional comparisons of the incremental costs and incremental effectiveness of AI and robotics in vascular interventional neurosurgery would provide a sound basis for justifying the adoption of new technologies in the surgical theater. Still, high initial costs should not deter the modern surgeon from understanding that investment in these technologies shows promise to fundamentally improve healthcare outcomes.

## Conclusions

Our review indicates that AI and robotics in endovascular neurosurgery have an extremely promising future, albeit with an unknown impact on interventional costs. Becoming familiar with this technology and understanding its intricacies and clinical applications can be beneficial for neurosurgeons and neurointerventionalists alike, as AI research and its clinical applications continue to grow and evolve. We hope to encourage not only neurosurgeons but also other physician specialties to become acquainted with these advances since they may become the standard for improved patient care in the future.

## References

[1] Davenport T, Kalakota R (2019). The potential for artificial intelligence in healthcare. Future Healthc J.

[2] Kaul V, Enslin S, Gross SA (2020). History of artificial intelligence in medicine. Gastrointest Endosc.

[3] Varghese J (2020). Artificial intelligence in medicine: chances and challenges for wide clinical adoption. Visc Med.

[4] Lane T (2018). A short history of robotic surgery. Ann R Coll Surg Engl.

[5] Beaman CB, Kaneko N, Meyers PM, Tateshima S (2021). A review of robotic interventional neuroradiology. AJNR Am J Neuroradiol.

[6] Riga CV, Bicknell CD, Hamady MS, Cheshire NJ (2011). Evaluation of robotic endovascular catheters for arch vessel cannulation. J Vasc Surg.

[7] Au S, Ko K, Tsang J (2014). Robotic endovascular surgery. Asian Cardiovasc Thorac Ann.

[8] Prevedello LM, Erdal BS, Ryu JL, Little KJ, Demirer M, Qian S, White RD (2017). Automated critical test findings identification and online notification system using artificial intelligence in imaging. Radiology.

[9] Ueda D, Yamamoto A, Nishimori M (2019). Deep learning for MR angiography: automated detection of cerebral aneurysms. Radiology.

[10] Titano JJ, Badgeley M, Schefflein J (2018). Automated deep-neural-network surveillance of cranial images for acute neurologic events. Nat Med.

[11] Yedavalli VS, Tong E, Martin D, Yeom KW, Forkert ND (2021). Artificial intelligence in stroke imaging: current and future perspectives. Clin Imaging.

[12] Rabinovich EP, Capek S, Kumar JS, Park MS (2020). Tele-robotics and artificial-intelligence in stroke care. J Clin Neurosci.

[13] Legeza P, Britz GW, Loh T, Lumsden A (2020). Current utilization and future directions of robotic-assisted endovascular surgery. Expert Rev Med Devices.

[14] Jones B, Riga C, Bicknell C, Hamady M (2021). Robot-assisted carotid artery stenting: a safety and feasibility study. Cardiovasc Intervent Radiol.

[15] George JC, Tabaza L, Janzer S (2020). Robotic-assisted balloon angioplasty and stent placement with distal embolic protection device for severe carotid artery stenosis in a high-risk surgical patient. Catheter Cardiovasc Interv.

[16] Pancaldi L, Dirix P, Fanelli A (2020). Flow driven robotic navigation of microengineered endovascular probes. Nat Commun.

[17] Gopesh T, Wen JH, Santiago-Dieppa D (2021). Soft robotic steerable microcatheter for the endovascular treatment of cerebral disorders. Sci Robot.

[18] Bao X, Guo S, Xiao N, Li Y, Yang C, Jiang Y (2018). A cooperation of catheters and guidewires-based novel remote-controlled vascular interventional robot. Biomed Microdevices.

[19] Morey JR, Zhang X, Yaeger KA (2021). Real-world experience with artificial intelligence-based triage in transferred large vessel occlusion stroke patients. Cerebrovasc Dis.

[20] Yahav-Dovrat A, Saban M, Merhav G (2021). Evaluation of artificial intelligence-powered identification of large-vessel occlusions in a comprehensive stroke center. AJNR Am J Neuroradiol.

[21] Block L, El-Merhi A, Liljencrantz J, Naredi S, Staron M, Odenstedt Hergès H (2020). Cerebral ischemia detection using artificial intelligence (CIDAI)-a study protocol. Acta Anaesthesiol Scand.

[22] Yao X, Mao L, Lv S, Ren Z, Li W, Ren K (2020). CT radiomics features as a diagnostic tool for classifying basal ganglia infarction onset time. J Neurol Sci.

[23] Rava RA, Peterson BA, Seymour SE (2021). Validation of an artificial intelligence-driven large vessel occlusion detection algorithm for acute ischemic stroke patients. Neuroradiol J.

[24] Bernard A, Comby PO, Lemogne B, Haioun K, Ricolfi F, Chevallier O, Loffroy R (2021). Deep learning reconstruction versus iterative reconstruction for cardiac CT angiography in a stroke imaging protocol: reduced radiation dose and improved image quality. Quant Imaging Med Surg.

[25] Kasasbeh AS, Christensen S, Parsons MW, Campbell B, Albers GW, Lansberg MG (2019). Artificial neural network computer tomography perfusion prediction of ischemic core. Stroke.

[26] Silva MA, Patel J, Kavouridis V (2019). Machine learning models can detect aneurysm rupture and identify clinical features associated with rupture. World Neurosurg.

[27] Faron A, Sichtermann T, Teichert N (2020). Performance of a deep-learning neural network to detect intracranial aneurysms from 3D TOF-MRA compared to human readers. Clin Neuroradiol.

[28] Zhu W, Li W, Tian Z (2020). Stability assessment of intracranial aneurysms using machine learning based on clinical and morphological features. Transl Stroke Res.

[29] Shimada Y, Tanimoto T, Nishimori M (2020). Incidental cerebral aneurysms detected by a computer-assisted detection system based on artificial intelligence: a case series. Medicine (Baltimore).

[30] Park A, Chute C, Rajpurkar P (2019). Deep learning-assisted diagnosis of cerebral aneurysms using the HeadXNet Model. JAMA Netw Open.

[31] Liu X, Feng J, Wu Z (2021). Deep neural network-based detection and segmentation of intracranial aneurysms on 3D rotational DSA. Interv Neuroradiol.

[32] Kim KH, Koo HW, Lee BJ, Sohn MJ (2021). Analysis of risk factors correlated with angiographic vasospasm in patients with aneurysmal subarachnoid hemorrhage using explainable predictive modeling. J Clin Neurosci.

[33] Kordzadeh A, Esfahlani SS (2019). The role of artificial intelligence in the prediction of functional maturation of arteriovenous fistula. Ann Vasc Dis.

[34] Fu F, Wei J, Zhang M (2020). Rapid vessel segmentation and reconstruction of head and neck angiograms using 3D convolutional neural network. Nat Commun.

[35] Lang S, Hoelter P, Schmidt M (2020). Evaluation of an artificial intelligence-based 3D-angiography for visualization of cerebral vasculature. Clin Neuroradiol.

[36] Heunis CM, S Uligoj F, Santos CF, Misra S (2021). Real-time multi-modal sensing and feedback for catheterization in porcine tissue. Sensors (Basel).

[37] Rava RA, Seymour SE, LaQue ME (2021). Assessment of an artificial intelligence algorithm for detection of intracranial hemorrhage. World Neurosurg.

[38] Teng L, Ren Q, Zhang P, Wu Z, Guo W, Ren T (2021). Artificial intelligence can effectively predict early hematoma expansion of intracerebral hemorrhage analyzing noncontrast computed tomography image. Front Aging Neurosci.

[39] Nawabi J, Kniep H, Kabiri R (2020). Neoplastic and non-neoplastic acute intracerebral hemorrhage in CT brain scans: machine learning-based prediction using radiomic image features. Front Neurol.

[40] Voter AF, Larson ME, Garrett JW, Yu JJ (2021). Diagnostic accuracy and failure mode analysis of a deep learning algorithm for the detection of cervical spine fractures. AJNR Am J Neuroradiol.

[41] Akiyama Y, Mikami T, Mikuni N (2020). Deep learning-based approach for the diagnosis of Moyamoya disease. J Stroke Cerebrovasc Dis.

[42] Yeo M, Tahayori B, Kok HK (2021). Review of deep learning algorithms for the automatic detection of intracranial hemorrhages on computed tomography head imaging. J Neurointerv Surg.

[43] Britz GW, Panesar SS, Falb P, Tomas J, Desai V, Lumsden A (2019). Neuroendovascular-specific engineering modifications to the CorPath GRX Robotic System. J Neurosurg.

[44] Cheung S, Rahman R, Bicknell C (2020). Comparison of manual versus robot-assisted contralateral gate cannulation in patients undergoing endovascular aneurysm repair. Int J Comput Assist Radiol Surg.

[45] Chi W, Liu J, Rafii-Tari H, Riga C, Bicknell C, Yang GZ (2018). Learning-based endovascular navigation through the use of non-rigid registration for collaborative robotic catheterization. Int J Comput Assist Radiol Surg.

[46] Miyachi S, Nagano Y, Kawaguchi R, Ohshima T, Tadauchi H (2021). Remote surgery using a neuroendovascular intervention support robot equipped with a sensing function: experimental verification. Asian J Neurosurg.

[47] Nogueira RG, Sachdeva R, Al-Bayati AR, Mohammaden MH, Frankel MR, Haussen DC (2020). Robotic assisted carotid artery stenting for the treatment of symptomatic carotid disease: technical feasibility and preliminary results. J Neurointerv Surg.

[48] Yamaki VN, Cancelliere NM, Nicholson P (2021). Biomodex patient-specific brain aneurysm models: the value of simulation for first in-human experiences using new devices and robotics. J Neurointerv Surg.

[49] Pannell JS, Santiago-Dieppa DR, Wali AR (2016). Simulator-based angiography and endovascular neurosurgery curriculum: a longitudinal evaluation of performance following simulator-based angiography training. Cureus.

[50] Asadi H, Kok HK, Looby S, Brennan P, O'Hare A, Thornton J (2016). Outcomes and complications after endovascular treatment of brain arteriovenous malformations: a prognostication attempt using artificial intelligence. World Neurosurg.

[51] Katsuki M, Kawamura S, Koh A (2021). Easily created prediction model using automated artificial intelligence framework (Prediction One, Sony Network Communications Inc., Tokyo, Japan) for subarachnoid hemorrhage outcomes treated by coiling and delayed cerebral ischemia. Cureus.

[52] de Jong G, Aquarius R, Sanaan B, Bartels RH, Grotenhuis JA, Henssen DJ, Boogaarts HD (2021). Prediction models in aneurysmal subarachnoid hemorrhage: forecasting clinical outcome with artificial intelligence. Neurosurgery.

[53] Asadi H, Dowling R, Yan B, Mitchell P (2014). Machine learning for outcome prediction of acute ischemic stroke post intra-arterial therapy. PLoS One.

[54] Weinberg JH, Sweid A, Sajja K (2020). Comparison of robotic-assisted carotid stenting and manual carotid stenting through the transradial approach. J Neurosurg.

[55] Du Y, Long Q, Guan B (2018). Robot-assisted radical prostatectomy is more beneficial for prostate cancer patients: a system review and meta-analysis. Med Sci Monit.

[56] Weisz G, Metzger DC, Caputo RP (2013). Safety and feasibility of robotic percutaneous coronary intervention: PRECISE (Percutaneous Robotically-Enhanced Coronary Intervention) Study. J Am Coll Cardiol.

[57] Wali AR, Brandel MG, Santiago-Dieppa DR (2018). Markov modeling for the neurosurgeon: a review of the literature and an introduction to cost-effectiveness research. Neurosurg Focus.

[58] Hartman M, Martin AB, Espinosa N, Catlin A, The National Health Expenditure Accounts Team (2018). National health care spending in 2016: spending and enrollment growth slow after initial coverage expansions. Health Aff (Millwood).

[59] Childers CP, Maggard-Gibbons M (2018). Estimation of the acquisition and operating costs for robotic surgery. JAMA.

[60] Lotan Y (2012). Is robotic surgery cost-effective: no. Curr Opin Urol.

